# The Treatment of Mitral Valve Disease—The Only Thing Constant is Change

**DOI:** 10.3390/biomedicines9020126

**Published:** 2021-01-28

**Authors:** Joseph Zacharias, Ernesto Greco

**Affiliations:** 1 Department of Cardiothoracic Surgery, Blackpool Teaching Hospitals NHS Foundation Trust, Whinney Heys Rd, Blackpool FY3 8NR, UK; drjzacharias@gmail.com; 2 Department of Clinical, Internal Medicine, Anaesthesiology and Cardiovascular Sciences, Sapienza University, 00161 Rome, Italy

The mitral valve is without doubt the part of the human body that is most under pressure. For every beat of the heart, the mitral valve has to open to let the blood into the most muscular chamber in the body, and then closes to withstand high systolic pressures that, during periods of exertion, can exceed over 200 mmHg.

It may be that these conditions lead to valve malfunction and the effects of malfunction are quickly felt by the individual. This valve has seduced anatomists, artists and physicians for a long time and rightly so, as it is a thing of beauty that most of us who operate on it are always humbled by. It is, hence, not surprising to note that this valve was one of the first to be dealt with by pioneering surgeons like Dr. Cutler in the early 1920s, nearly a century ago [1]. Progress in the treatment of the mitral valve has always been sporadic, with short periods of rapid therapeutic advances followed by longer periods of wider dissemination in the larger surgical community. After description and acceptance of the closed mitral valvotomy [2] the next big advances occurred in the late 1950s and early 1960s, with surgeons presenting techniques to repair or replace the mitral valve using the cardiopulmonary bypass machine. The initial enthusiasm was to replace the mitral valve with a mechanical prosthesis popularised by Dr. Starr [3]. Even though the initial results were rewarding, a need for valves made from tissue rather than metal led to a large increase in the popularity of replacement valves using tissue stabilisation techniques put forward by Dr. Carpentier [4]. Repairing the mitral valve had predated attempts to replace it but it took to the late 1970s and 1980s for mitral valve repair techniques to be accepted widely and become standardised by the pioneering efforts of Dr. Duran and Dr. Carpentier, among others [5]. Gradually, safe and reproducible techniques for repairing the mitral valve spread gradually across the cardiac surgical world and, around that time, percutaneous balloon valvotomy was also gaining popularity in selected cases of non-calcified mitral stenosis [6]. Despite these advances in the management of mitral valve disease, most patients were managed medically and presented to surgery late, and benefits of surgery were compromised due to the preoperative state of many patients. This was impacted by many excellent teams showing the possible normal life expectancy of patients referred early for mitral valve repair. Dr. Frater’s and Dr. David’s work in increasing the use of artificial chords led to more mitral valves being repaired and a larger group of surgeons being able to offer these procedures into the new century [7,8]. The next big step in the field was to move away from a sternotomy approach to a non-sternotomy approach to the mitral valve.

Of note, the original mitral valve replacements done by Dr. Starr were using a right lateral thoracotomy and the closed mitral procedures, through a left lateral thoracotomy. The real challenge for the generation of surgeons that followed was to reduce the trauma to the chest wall. Dr. Vanermen led the field in using an endoscope and the endoclamp to reduce the size of the mini-thoracotomy, Dr. Mohr led the field to train many young surgeons using a minithoracotomy and Dr. Chitwood championed the use of the robotic console and the external cross clamp on the aorta to achieve excellent non sternotomy approaches to deal with the mitral valve, and all three of them published their excellent results in the early decade of this century [9,10,11]. This led to a large interest across the globe and the past 20 years have led to a gradual and safe adoption of these techniques by many surgeons and their teams [12,13,14,15]. Following the successful attempts to deal with the aortic valve with trans-catheter techniques, there has been a widespread interest in finding both repair and replacement options for the mitral valve with an avoidance of cardiopulmonary bypass and any incisions on the chest. The largest experience worldwide is with the Mitraclip^®®®^ device and it appears to give good short-term results in selected patients.

This edition captures the experience of some world leaders in other new technologies already available and on the horizon. As mentioned in the run-up, there has always been many techniques available and, over time, some have stood the test of time and have been added to the armamentarium available for clinicians dealing with this crucial, complex and beautiful valve.

It is said that those who forget the lessons of history are likely to repeat the mistakes of the past. What we surgeons have learned about this particular valve over the past 100 years, is that the pathology affecting the mitral valve is varied and quite complex. It is unlikely that one device or one technique will deal with the many complex conditions, and there will need to be an acceptance, that the therapy of the mitral valve is likely to need a multidisciplinary approach. Repairing the mitral valve to anatomically work as a bi-leaflet valve along with a ring to fix the annulus, provides the best long-term results by achieving a life expectancy for patients that match age matched cohorts. As we pick up patients earlier in the disease pathway, it is unlikely that an approach that disrupts the chest wall is likely to be tolerated both by the asymptomatic young or the frail elderly patient.

Trans-catheter approaches are likely to have a role in the very sick and the very frail as most surgical options are unlikely to provide the short-term benefit needed. An endoscopic approach to treat the pathology of the mitral valve is likely to be the most cost-effective treatment with the excellent long-term benefits with the least short-term disruption to patients [16]. The challenge for cardiac surgeons will be the wider dissemination of these intricate techniques in the current climate of reduced training time and a societal intolerance to learning curves in surgical interventions. We will need to revisit the cycles of the past where safe and slow dissemination was achieved by the close collaboration of expert surgeons who also need to be mentors and teachers [17].

This focus issue is a start, to try and capture the experience of experts. Interested doctors would benefit from arranging visits or meetings with these experts to try and understand how specific techniques and technologies can be evaluated in their own hospitals. It is crucial that dealing with patients with mitral pathology will require a multidisciplinary team approach. The individuals in the team will need to know their strengths and most importantly their weak spots. A strong team is one where individuals align together to put their strengths forward together to reduce the weak areas. In dealing with the mitral valve all approaches will have weak spots and we need to combine our efforts with a mixture of humility and curiosity to achieve the best results for our masters, the patient.
